# Rapid sample preparation with Lyse-It® for *Listeria monocytogenes* and *Vibrio cholerae*

**DOI:** 10.1371/journal.pone.0201070

**Published:** 2018-07-25

**Authors:** Tonya M. Santaus, Shan Li, Paula Ladd, Amanda Harvey, Shannon Cole, O. Colin Stine, Chris D. Geddes

**Affiliations:** 1 University of Maryland, Baltimore County, Chemistry and Biochemistry Department, Baltimore, MD, United States of America; 2 Institute of Fluorescence, University of Maryland, Baltimore County, Baltimore, MD, United States of America; 3 University of Maryland School of Medicine, Epidemiology and Public Health Department, Baltimore, MD, United States of America; Institute of Materials Science, GERMANY

## Abstract

Sample preparation is a leading bottleneck in rapid detection of pathogenic bacteria. Here, we use Lyse-It^®^ for bacterial cellular lysis, genomic DNA fragmentation, and protein release and degradation for both *Listeria monocytogenes* and *Vibrio cholerae*. The concept of Lyse-It^®^ employs a conventional microwave and Lyse-It^®^ slides for intensely focused microwave irradiation onto the sample. High microwave power and a <60 second irradiation time allow for rapid cellular lysis and subsequent intracellular component release. The pathogenic bacteria are identified by quantitative polymerase chain reaction (qPCR), which subsequently demonstrates the viability of DNA for amplification post microwave-induced lysis. Intracellular component release, degradation, and detection of *L*. *monocytogenes* and *V*. *cholerae* has been performed and shown in this paper. These results demonstrate a rapid, low-cost, and efficient way for bacterial sample preparation on both food and water-borne Gram-positive and -negative organisms alike.

## Introduction

While DNA molecular detection methods today are modestly rapid, it is slow and complex sample preparation that hinders rapid point-of-care testing. There are numerous kits that aid in sample cellular lysing like OmniLyse^®^, various lysis buffers, CelLytic^TM^ B Plus Kit, and GenElute^TM^, just to name a few [1, 2]. However, the set back to these kits is that DNA fragmentation and/or protein degradation is a second separate step following the initial cellular lysis. Therefore, having a one-step protocol that performs both cellular lysis and DNA fragmentation/ protein release and degradation is pivotal to rapid diagnostics. In addition, these kits for both cellular lysis and DNA fragmentation often have limited shelf lives, require refrigeration, and a cold chain for transportation. In this paper, we discuss the Lyse-It^®^ technology utility on food and water-borne Gram-positive and Gram-negative organisms. These are bacteria where rapid detection is needed and where current sample preparation cost is a hindrance in low-resource settings.

*Listeria monocytogenes* and *Vibrio cholerae* are food and water-borne pathogenic bacteria respectively, which can cause significant harm to those who ingest them. *L*. *monocytogenes* primarily affects the elderly, those with a compromised immune system, and pregnant women [3–7]. According to the Center for Disease Control and Prevention, there are approximately 1,600 people infected each year of which 16% of cases are fatal in the US[8]. Individuals who contract *V*. *cholerae* primarily live in locations of poor sanitation and water quality. According to the World Health Organization, there are 1.4 to 4.3 million cases and 28,000 to 142,000 deaths worldwide due to *V*. *cholerae* every year [9–11]. With these statistics, it is not surprising that there is an urgent need for a rapid bacterial lysing and detection method.

Lyse-It^®^ is a new commercialized technology that combines rapid cellular lysing with DNA/RNA fragmentation, protein release and degradation on demand in one simple, low-cost step. The technology incorporates equilateral gold bow-tie geometries vapor deposited on to 3” x 1” glass slides and held stationary in a conventional microwave cavity with a non-microwave absorbing sample-holding block (**S1 Fig**). The theory behind the gold bow-tie geometry used, the calculations demonstrating the microwave focusing, and the subsequent thermal gradients in Lyse-It^®^ have been extensively reviewed [12–19]. Lyse-It^®^ is very different from conventional microwave irradiation in that the gold bow-tie geometry focuses the microwaves directly to the center of the sample allowing for a rapid increase in electromagnetic energy and temperature to the sample. Highly focused microwave irradiation creates large thermal and convection gradients throughout the sample of cells, bacteria, or viruses that far exceeds the mechanical strength of any cell membrane allowing for the entire sample to be exposed to intense microwaves. Interestingly, the mechanism(s) for cellular lysis and later biomolecule degradation are not predominantly thermal, as the sample increases above room temperature only to the range of ≈ 30–50°C. This technology can be used with various bacterial suspension media like phosphate buffered saline, Tris-EDTA, and deionized water while also being applicable in clinical settings with samples such as blood, vaginal mucus, feces etc. as well as with environmental samples, such as soil [12, 20–24]. By utilizing Lyse-It^®^, cellular lysis, DNA fragmentation, and both protein release followed by degradation at later times is achieved in under 60 seconds for *L*. *monocytogenes* and *V*. *cholerae*.

## Materials and methods

### Bacterial strains and growth conditions

*L*. *monocytogenes* and *V*. *cholerae* were grown on sheep blood agar and Thiosulfate Citrate Bile Sucrose (TCBS) agar plates respectively in an air environment for 24 hours at 37°C with 2% CO_2_. Colonies were suspended in DI water and the concentration was determined on a Varian Eclipse 60 UV-Spectrophotometer at 600 nm following McFarland standards. All experiments were performed using stock solutions of 10^8^ colony-forming units (cfu/mL).

### Osmotic lysing

*L*. *monocytogenes* and *V*. *cholerae* stock solutions were osmotically lysed on the benchtop at room temperature for up to 2.5 weeks. Following osmotic lysing times ranging between immediately and 2.5 weeks, cell suspensions were ethanol precipitated and the DNA rehydrated. Ethidium bromide stained gel electrophoresis was performed to visualize double-stranded DNA fragmentation. Osmotic lysing was undertaken as a control experiment to demonstrate the extensive time required for cellular lysis and DNA fragmentation to occur as compared to the Lyse-It^®^ technology.

### Conventional heating and microwave irradiation

Stock *L*. *monocytogenes* and *V*. *cholerae* solutions (2mL) were conventionally heated in a glass vial capped with a thermometer inset on a hot plate for 1 minute at temperatures ranging between 40°C and 80°C ± 1°C. Stock bacterial solutions (1mL) were microwave irradiated in a 900W Frigidaire microwave for 1 min at microwave powers between 10% and 50% which are equivalent to 90W and 450W respectively. Following conventional heating and microwave irradiation, samples were cooled to room temperature for 1 hour allowing for DNA re-hybridization occur and so that cellular cold shock would not occur with ethanol precipitation. Lysed and healthy (un-lysed) samples were ethanol precipitated with cold ethanol (-15° to -25°C) and the DNA was rehydrated in DNA rehydration buffer, followed by gel electrophoresis on ethidium bromide stained 1.5% gels. SYPRO Ruby stained sodium-dodecylsulfate gel electrophoresis (SDS PAGE) was used to visualize protein release post lysing.

### Aligent 2100 Bioanalyzer

In order to analyze and quantify small DNA fragments, *L*. *monocytogenes* samples were run on the Aligent Bioanalyzer 2100 system utilizing the Aligent DNA 1000 Kit (Aligent). The Bioanalyzer analysis was performed at the Institute of Marine and Environmental Technology Bio Analytical Services Lab in Baltimore, MD. *V*. *cholerae* samples were not run on the Bioanalyzer as clear agarose gels were sufficient for visualization of DNA extraction and fragmentation. *L*. *monocytogenes*, at the microwave conditions described in this paper, only allow for low concentrations of DNA to be released, thus a more sensitive detection method was used for DNA extraction and fragmentation analysis.

### Dynamic Light Scattering (DLS) and Transmission Electron Microscopy (TEM)

DLS and TEM were performed to investigate cellular lysis following conventional heating and microwave irradiation. Post conventional heating and microwave irradiation procedures described above, lysed bacterial samples were executed on a Malvern Zetasizer Nano series ZS dynamic light scattering instrument. The sample refractive index was 1.330 and the absorbance at 632 nm was 0.062. Cellular images of the un-lysed and lysed bacteria were taken on a FEI Morgagni and Zeiss-1OCA Transmission Electron Microscope. 10 μL of bacterial cells were incubated onto carbon/copper TEM grids, stained with 10 μL of 1% uranyl acetate, rinsed with 10 μL DI water, and allowed to dry overnight. Images were subsequently chosen which showed membrane disruption, as compared to completely lysed images, which show nothing visual.

### Polymerase Chain Reaction (qPCR)

Quantitative polymerase chain reaction (qPCR) was performed in the Department of Epidemiology and Public Health at the University of Maryland School of Medicine. Samples were lysed as described above and centrifuge filtered using 0.22μm centrifugal filter units at 6000 rpm for 5 minutes. The filtered solution containing the DNA was stored in a -20°C freezer until analysis. An ABI QuantStudio 3 qPCR was used with the following primers for *L*. *monocytogenes* and *V*. *cholerae* respectively. *L*. *monocytogenes* forward primer 5’-GCAATTTCGAGGCCTAACCTA and reverse primer 5’–ACTGCGTTGTTAACGTTTGA– 3’ amplifying part of the L-hemolysin gene. *V*. *colerae* forward primer 5’-CTCAGACGGGATTTGTTAGGCACG-3’ and reverse primer 5’-TCTATCTCTAGCCCCTATTACG-3’ for amplification of the ctxA gene using a SYBR green assay.

## Results

### Minimal cellular lysing and DNA fragmentation from osmotic conditions

To investigate potential osmotic lysing effects prior to microwave irradiation on Gram-positive and -negative organisms, we first determined the time required for cellular lysis and in particular, DNA fragmentation to occur. We examined a range of time intervals from a few hours to as long as 2.5 weeks at room temperature and concluded that cellular lysis and visible DNA fragmentation were seen at 48 hours for *V*. *cholerae* and approximately 1 week for *L*. *monocytogenes* (**S2 Fig**). In general, we observed that osmotic lysis is a very slow process and DNA fragmentation does not occur within the time-period of our experiments, which is approximately 3 total hours from time of bacterial solution preparation, cooling, to sample preparation for gel electrophoresis. Subsequently, we are confident that cellular lysis and DNA fragmentation is minimal osmotically and thus does not affect the degree of cellular lysis and DNA fragmentation from utilizing the Lyse-It^®^ microwave technology, i.e. they occur on completely different time scales.

### Rapid temperature increase, cellular lysis, and DNA fragmentation is associated with microwave focusing of the Lyse-It^®^ system

In previous studies it was both theoretically and experimentally shown that the equilateral “bow-tie” geometry of the Lyse-It^®^ slide focuses microwaves leading to a significant thermal gradient and cavitation between the apexes [12, 15–19]. These thermal gradients (expansion of water) were shown to create enormous stress on cell walls, resulting in complete membrane collapse and release of the intact cellular materials. In those previous studies, the practical utility of Lyse-It^®^ to both Gram-negative and -positive organisms was not demonstrated, which is the crux of this paper. In addition, a comparison between standard microwave heating versus Lyse-It^®^ has not been explored. To *further* investigate the temperature differences between standard microwave heating and microwave heating with Lyse-It^®^, temperatures post microwave irradiation of *V*. *cholerae* and *L*. *monocytogenes* were taken using a Traceable^®^ Dual Laser IR Thermometer. We observed that, as the overall wattage of the microwave increases, the temperature logically increases, but with the Lyse-It^®^ technology, the temperature was higher overall than that of standard microwave irradiation (**Table 1**). These results confirm the theoretical studies and other studies performed on sexually transmitted infections[25], that when microwaves are focused using the Lyse-It^®^ technology, there is a notable increase in the total temperature of the sample, and not simply at the apex regions of the sample.

**Table 1:** Microwave irradiation temperatures.

Microwave Wattage	Standard Microwave Irradiation (°C)	Microwave Lysing with Lyse-It^®^ (°C)
***Vibrio cholerae* (60 seconds)**		
90	44.2	62.1
180	37.8	54.1
270	45.2	68.1
360	57.0	60.3
450	77.0	75.8
***Listeria monocytogenes* (60 seconds)**		
90	28.6	27.4
180	39.2	37.6
270	49.4	49.4
360	56.8	58.8
450	64.8	67.2

Post microwave irradiation temperature of *Vibrio cholerae* and *Listeria monocytogenes* with and without the use of Lyse-It^®^. As the microwave power increases the temperature increases.

**Fig 1:**
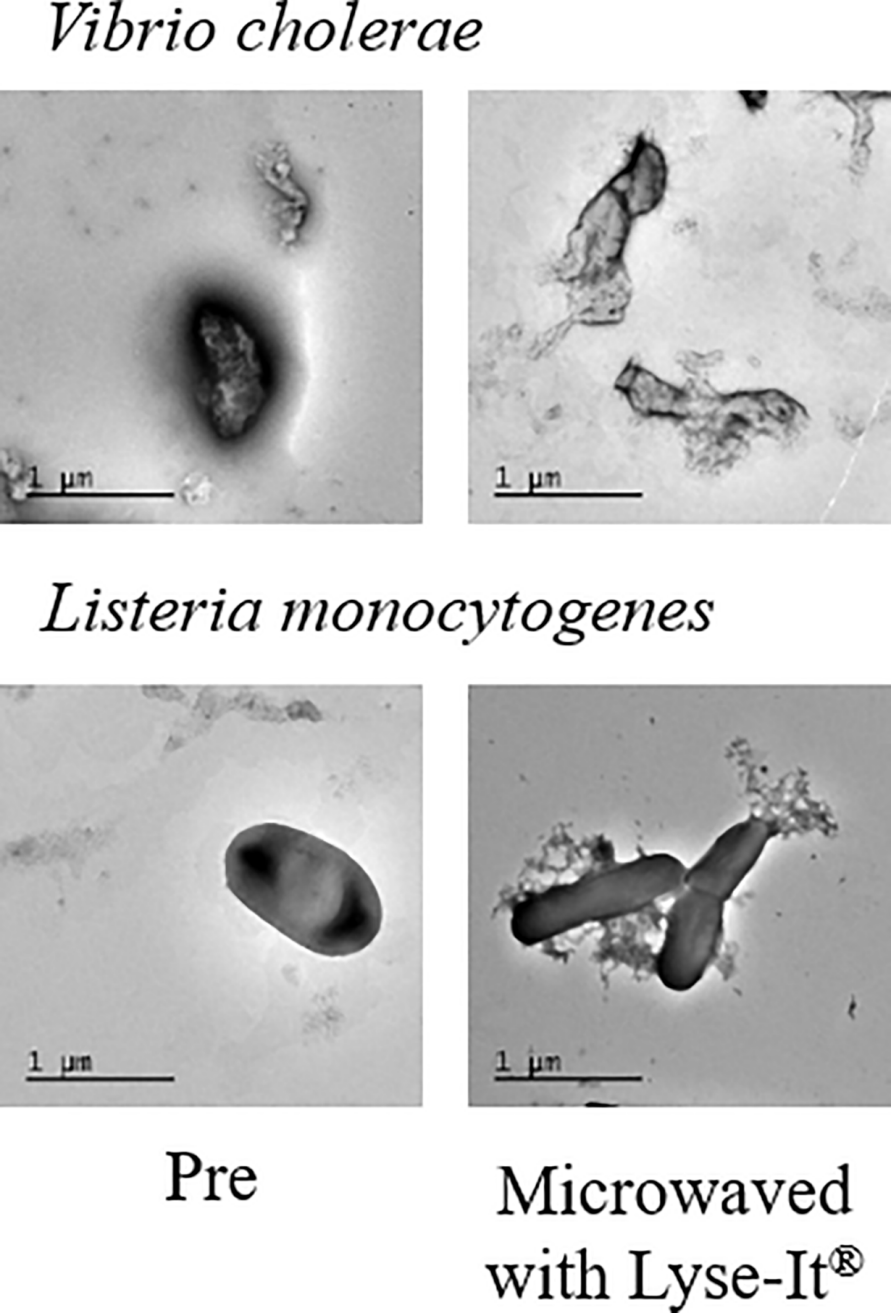
TEM images representing microwave cellular lysis. **Left:** Before microwave heating. **Right:** After microwave heating. *V*. *cholerae* was microwaved at 30% power (270 watts) for 60 seconds. *L*. *monocytogenes* was microwaved at 50% power (450 watts) for 60 seconds.

**Fig 2:**
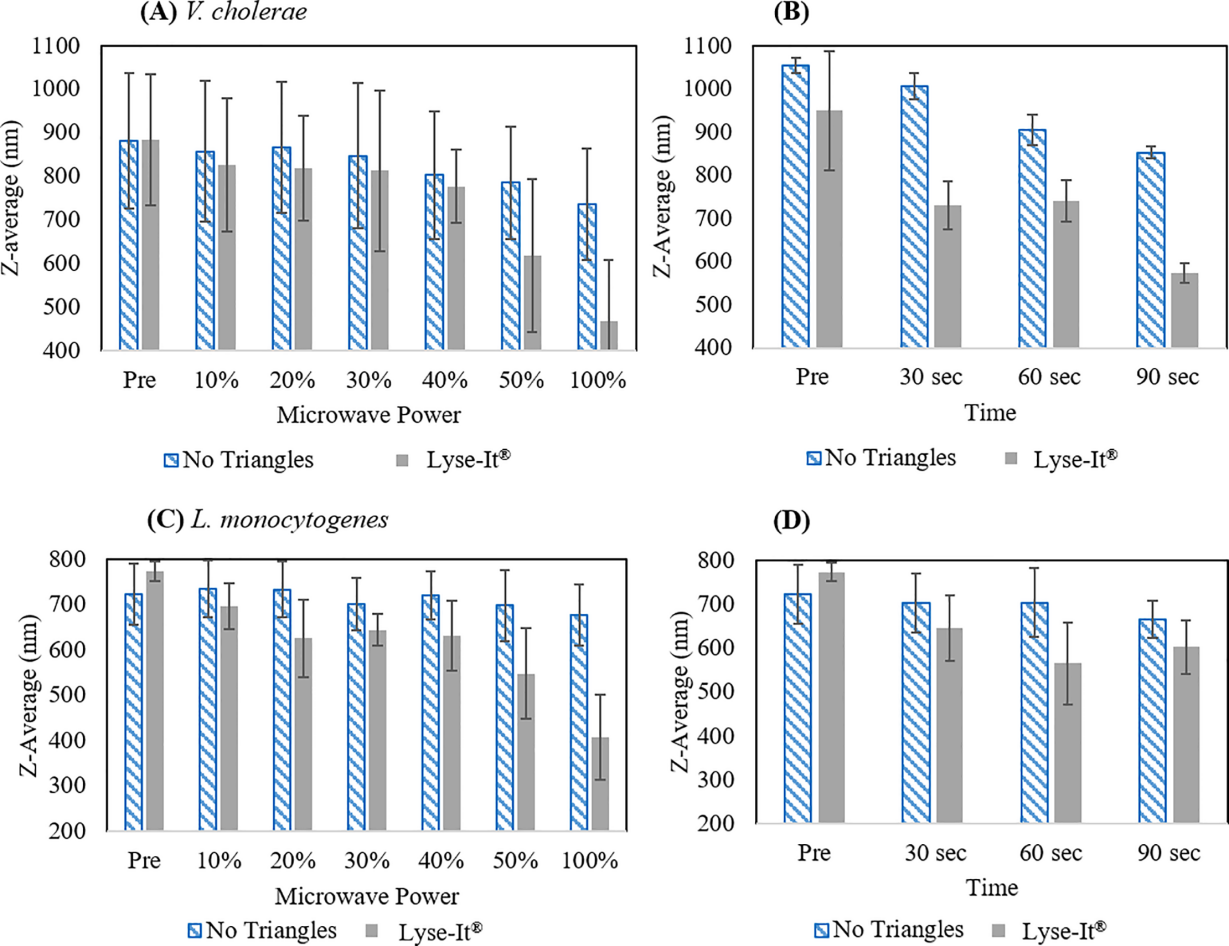
Dynamic Light Scattering (DLS) of microwave lysed *V*. *cholerae* and *L*. *monocytogenes* with and without Lyse-It^®^. **(A)** *V*. *cholerae* microwave irradiated for 60 seconds at varying powers and **(B)** microwaved for varying times at 30% power. **(C)** *L*. *monocytogenes* microwave irradiated for varying powers for 60 seconds and **(D)** microwaved at 50% power for varying times. It is clear that when Lyse-It^®^ is used, there is a decrease in the overall cellular size indicating that cellular lysis is occurring and is more efficient with Lyse-It^®^ as compared to standard microwave heating.

Following temperature studies, cellular disruption of *V*. *cholerae* and *L*. *monocytogenes* was investigated. Due to the inherent membrane structural differences of Gram-negative and -positive bacteria [26, 27], different microwave cavity powers were used so that TEM images could be taken of incomplete cellular lysis instead of complete cellular breakdown where very little would be visible on the images [12]. As described above, Lyse-It^®^ microwave irradiated cells were stained and imaged using TEM (**Fig 1**). *V*. *cholerae* and *L*. *monocytogenes* were microwave irradiated for 60 seconds at 30% and 50% power (270 and 450W) respectively. Healthy intact bacteria were shown to have a size of 1 μm with intact and smooth cellular definition. Post microwave irradiation, the cells of both bacteria are significantly distorted with a mix of cellular debris and intracellular components seen surrounding the cells. These images visually show the rapid cellular disruption from using Lyse-It^®^ technology. Further confirmation of cellular disruption can be seen from Dynamic Light Scattering (DLS) *z*-average analysis. DLS z-averages of standard microwave irradiation compared to Lyse-It^®^ were performed by varying the power and the time of the irradiation (**Fig 2**). In general, as the microwave power or time increases, the z-average decreases indicating that healthy bacterial cells, 700–900 nm, are broken down and intracellular components and debris are being detected through scattering. Giving us further confidence in the data, we see systematic decreases over time with an increased energy (microwave power multiplied by time) in the average size of the lysate particles. The range of sizes and aggregation of cellular debris for an average of trials are the contributors to the error bars post microwave irradiation. However, in every sequential trial, as the microwave power or time increased there was a noticeable and systematic decrease in the reported z-average. Interestingly, smaller z-average sizes are seen consistently with the use of Lyse-It^®^ as compared to standard microwave irradiation, further validating that Lyse-It^®^ rapidly lysis both Gram-positive and -negative organisms. It is also interesting to compare both TEM images of lysates with that of the DLS results. Although most TEM images for samples lysed at 30% power for 60 seconds routinely showed the destruction of all cells, this may not always be true, as shown in **Fig 2**where total destruction does not appear to be achieved for some cells.

**Fig 3:**
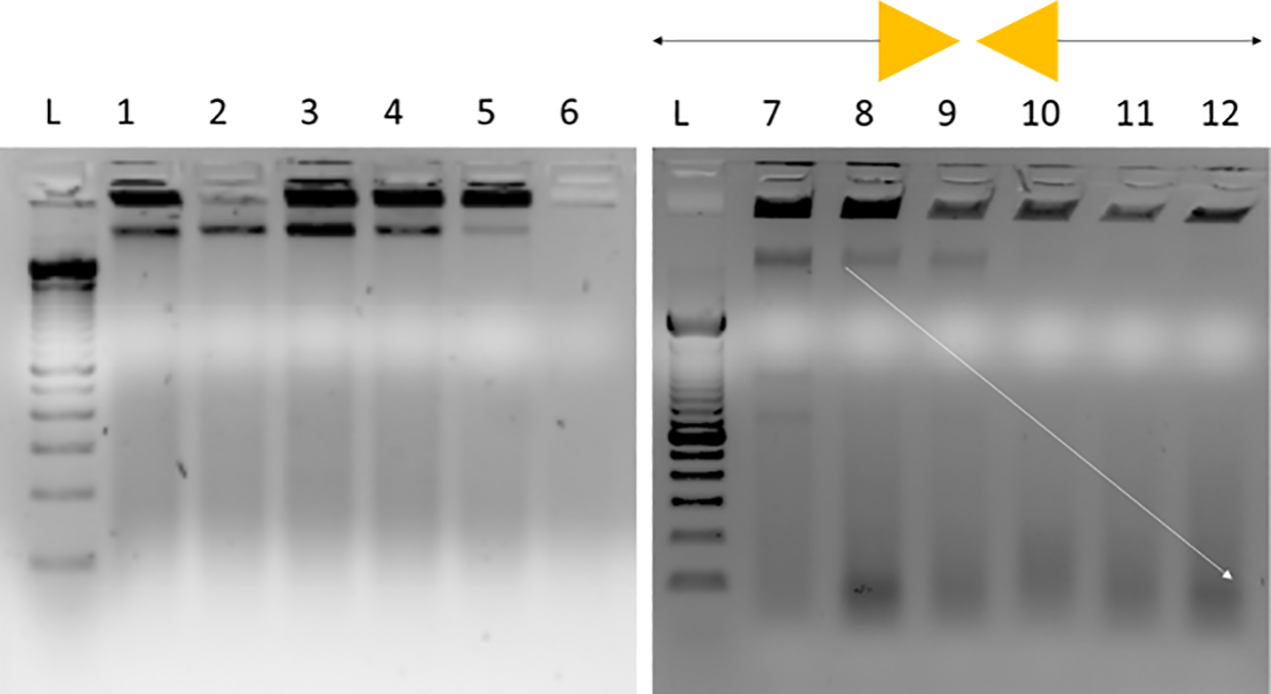
Microwave cellular lysis visualization through gel electrophoresis. Microwave cellular lysis of [10^8^ cfu] *V*. *cholerae* at 30% power (270 watts), (**Left**) Standard microwave heating, (**Right**) Microwave heating with Lyse-It^®^. L: 100 bp ladder, 1: Pre *V*. *cholerae* microwave irradiation, 2–6: 15–75 seconds no Lyse-It^®^, 7: Pre *V*. *cholerae* microwave irradiation, 8–12:15–75 seconds Lyse-It^®^.

**Fig 4:**
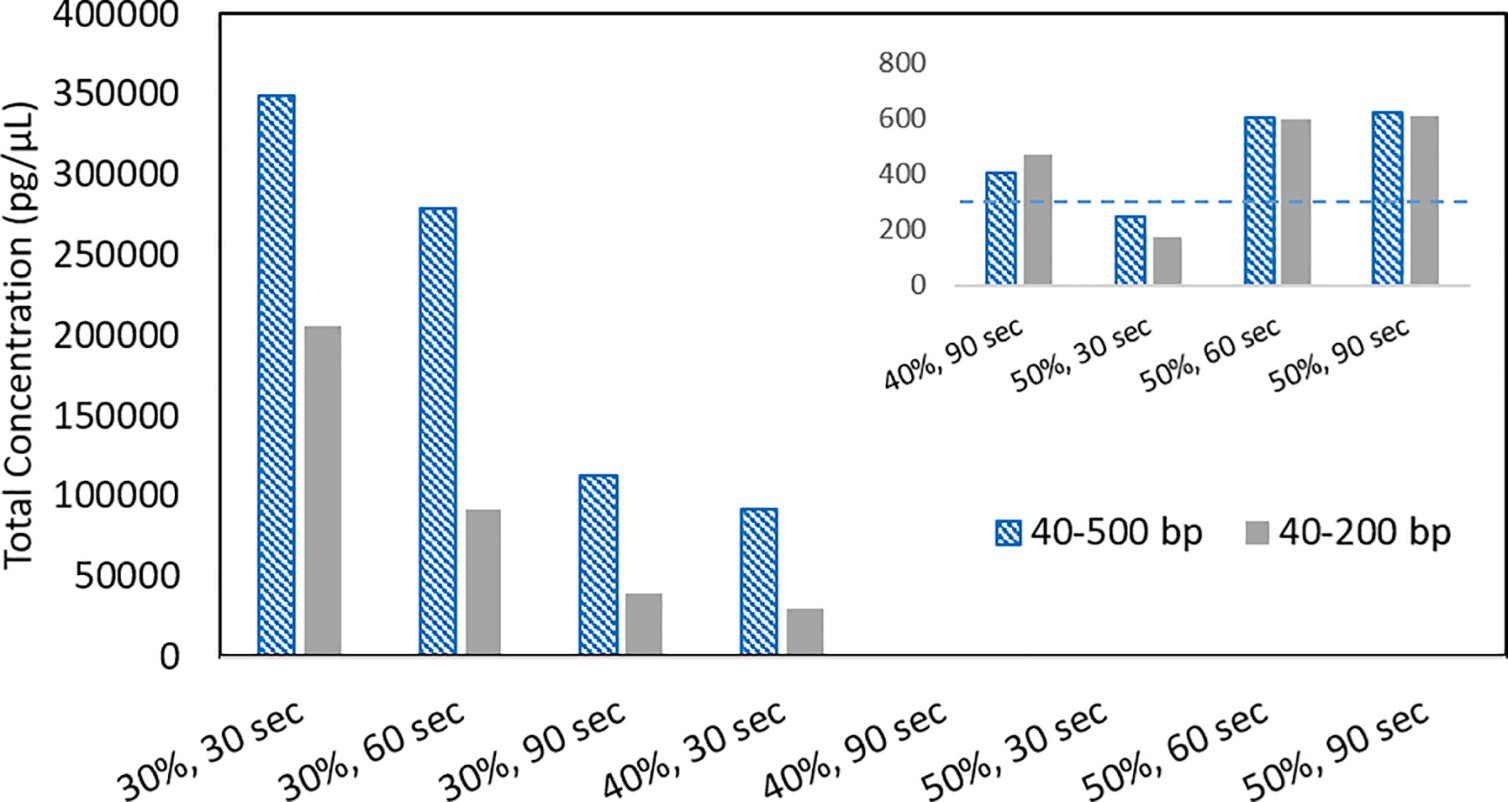
Microwave cellular lysis visualization using a mini-gel instrument. Aligent 2100 Mini-gel Bioanalyzer analysis of *L*. *monocytogenes* lysate microwave lysed with Lyse-It^®^. As the power and the time of the microwave increases the overall DNA concentration decreases indicating more continues shearing (cutting) of genomic DNA, to smaller and smaller sizes.

To determine the degree of DNA fragmentation through focused microwave irradiation, two forms of DNA fragmentation analysis were performed: standard agar gel electrophoresis and the Aligent Bioanalyzer mini-gel system. Concentrated *V*. *cholerae* (10^8^ cfu) was microwave irradiated with and without Lyse-It^®^ and the lysate prepared for gel electrophoresis as described in the methods section. The microwave power was kept constant (30%, 270W) and the time varied to investigate the effects of irradiation time on bacterial cells. Standard microwave irradiation (**Fig 3**left) shows that some DNA is fragmented, but a substantial amount of genomic DNA and cellular debris remains in the wells even after 75 seconds of irradiation. Conversely, with the use of Lyse-It^®^, there is a systematic increase in DNA fragmentation as seen below 500 bp and a decrease in the amount of genomic DNA in the wells as irradiation time increases (**Fig 3**right). Further investigation of Lyse-It^®^ time and power effects on DNA fragmentation was performed using a Bioanalyzer mini-gel system. *L*. *monocytogenes* (10^8^ cfu) was microwave irradiated with Lyse-It^®^ for varying powers and times, 30–50% and 30–90 seconds respectively (**Fig 4**). Over-all, as the power increases the total concentration of detected DNA between 40 bp and 500 bp decreases. These results indicate that genomic DNA is being fragmented into smaller and then smaller pieces over time, which is ideal for common detection platforms used today such as qPCR. However, looking at the inset of **Fig 4**, there is a systematic decrease in DNA size, until approximately 300 bp mean size is reached. However, from the original concentration of 350,000 pg/μL at low power, the concentration of DNA has dropped approximately 583 fold, which is believed to be well past the dynamic detection range of the instrument. This suggests that the DNA should simply decrease in base-pair size over both power and time, if the Bioanalyzer were able to measure it. These results from both gel systems demonstrate that DNA fragment size and concentration are directly a function of microwave power and time. Most importantly, this data shows that the Lyse-It^®^ technology can be tuned for DNA fragment size and concentration, which is ideal for use with the differing DNA amplification platforms.

### DNA is released and fragmented following Lyse-It^®^ is viable for qPCR detection

**Fig 5:**
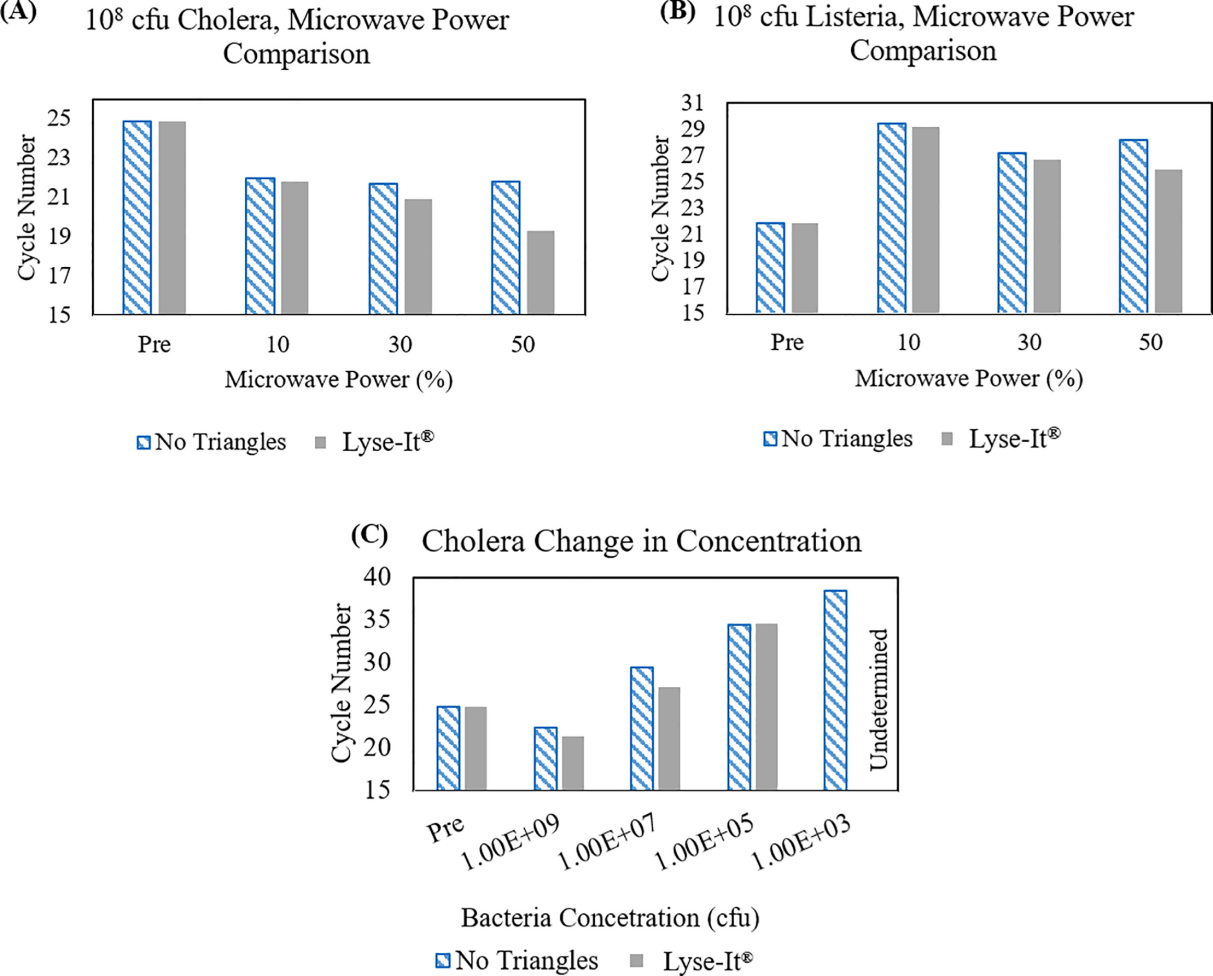
qPCR for the detection of *V*. *cholerae*. qPCR cycle number for increasing microwave irradiation powers and decreasing *V*. *cholerae* concentrations. **(A/B)** Decreasing cycle numbers with the use of triangles indicated an increase in released target DNA. **(C)** Increasing cycle numbers for decreasing concentrations of target DNA.

Following conformation that Lyse-It^®^ rapidly and effectively lysis cells and fragments DNA, we wanted to validate that the extracted DNA is indeed suitable for detection on a standard qPCR platform. Therefore, we examined lysing effects on both *V*. *cholerae* and *L*. *monocytogenes* DNA with and without Lyse-It^®^. *V*. *cholerae* and *L*. *monocytogenes* samples (10^8^ cfu) were prepared and irradiated for 60 seconds at 10, 30, and 50% power. Samples were centrifuge filtered to separate out cells and cellular debris from DNA and the DNA analyzed by qPCR (**Fig 5**). Cells that were not microwave irradiated, defined as *Pre*, were also subjected to the same filtration protocol as microwaved cells. In the case of *V*. *cholerae*, the cycle number for non-lysed cells was less than that of DNA extracted and filtered from lysed cells due to all available cells being 100% lysed through qPCR in the Pre sample. As the microwave power increases, more DNA is released from the cells resulting in a lower cycle number. Comparing standard microwave irradiation to focused microwaves, more DNA is detected with the use of Lyse-It^®^, especially at higher microwave powers. The significance of **Fig 5A** and **5B**, cannot be overstated. Typically, sample lysis and DNA fragmentation is performed in two discreet steps, using two separate buffer and enzyme solutions respectively. **Fig 5**shows that qPCR viable DNA can be obtained using Lyse-It^®^ in one <60 second step, using one low-cost sample chamber.

Following experimentation on varying microwave powers, the concentration of bacteria was also explored for DNA viability and detection. Serial dilutions of *V*. *cholerae* were prepared and exposed to microwave irradiation (**Fig 5C**). As expected, higher concentrations of *V*. *cholerae* had fewer cycle times, and that using Lyse-It^®^ also reduced the number of cycles as compared to standard microwave heating without Lyse-It^®^. An undetermined qPCR result was obtained for a concentration of 10^2^ cfu using Lyse-It^®^. We attribute this result to two primary factors: a higher ratio of PCR inhibitors to a low concentration of detectable specific DNA fragments and the concentration dependent reaction of hybridization of primer to target DNA during qPCR. These results demonstrate that genomic DNA from Gram-positive and -negative organisms is suitable for amplification and detection on qPCR platforms, post Lyse-It^®^.

### Lyse-It^®^ extracts out more proteins compared to standard microwave heating

**Fig 6:**
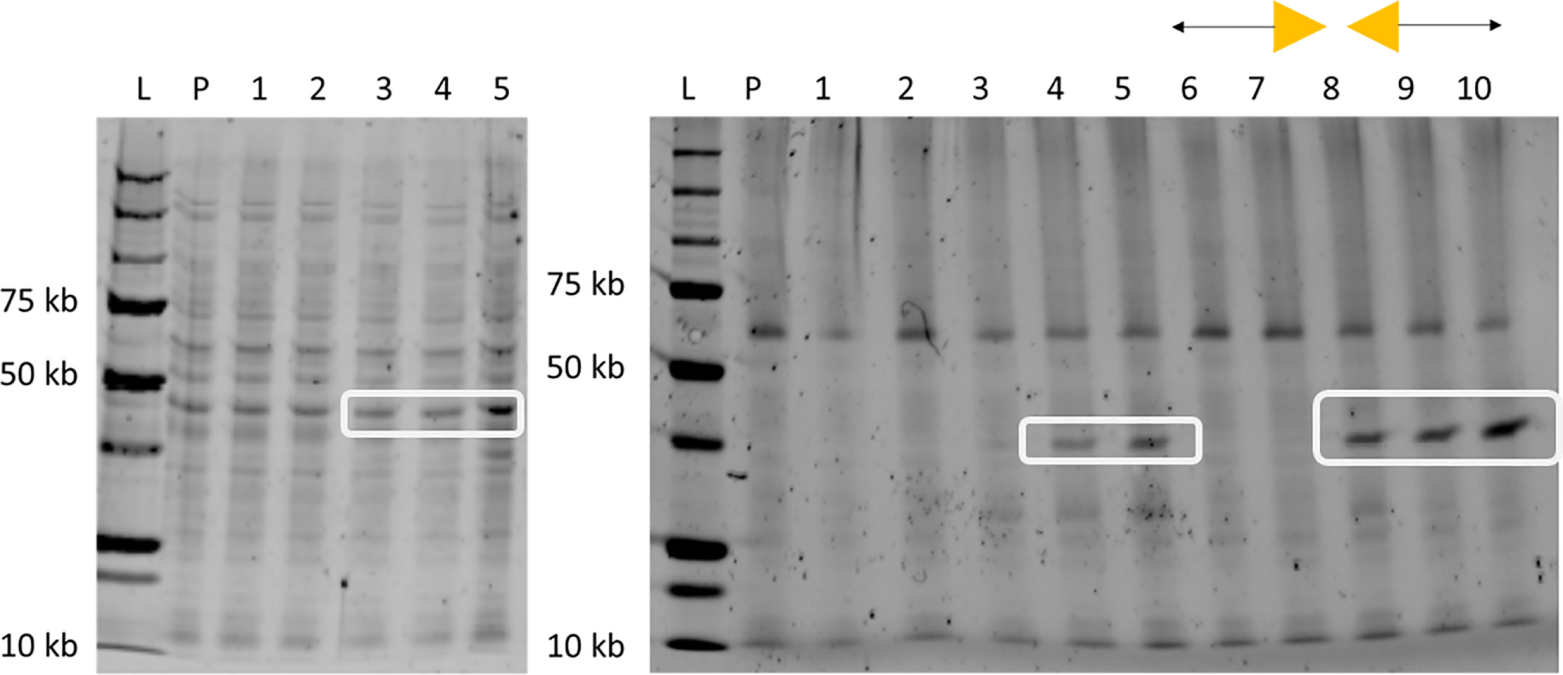
SDS PAGE of *V*. *cholerae*. **(A)** *V*. *cholerae* conventionally heating and microwave irradiated. L: Ladder, P: Pre-Lyse *V*. *cholerae*. **Left:** Conventionally heating. Lanes 1–5: 40°C—80°C, **Right:** Microwave irradiated. Lanes 1–5 No Lyse-It^®^ 10–50% power, 60 seconds respectively. Lanes 6–10: Lyse-It^®^ 10–50% power, 60 seconds respectively. **Highlighted regions** demonstrate that more protein is released with Lyse-It^®^.

We further investigated the effects of microwave-based lysing on protein extraction and subsequent degradation, given the widespread use of cellular lysing in proteomics. To explore this, we compared conventional heating methods to microwave irradiation with and without Lyse-It^®^. *V*. *cholerae* solutions (10^8^ cfu/mL) were heated conventionally, microwave irradiated, or irradiated using Lyse-It^®^. The conventional heating method did not result in more protein being released or substantially degraded as the sample temperature increased (**Fig 6 Left**). In essence, we can consider this a control sample to which to compare the effects of microwave irradiation. Conversely, standard microwave irradiation releases proteins from the cells, but also degrades some of the low molecular weight proteins as power increases. However, comparing standard microwave heating to Lyse-It^®^, we observe that lower molecular weight proteins are indeed released with lower powers using the focused microwaves and *in a greater abundance* (**Fig 6 Right and highlighted regions**). These protein results indicate that not only is DNA being released, but other intracellular components including proteins are being released as well at early (short) lysing times, thereby confirming that the Lyse-It^®^ technology is a multifunctional technique for the release of a variety of cellular components.

### Lyse-It^®^ slides cannot be reused

Finally, we questioned whether the Lyse-It^®^ slides could be reused after use. We found that after a modest irradiation time of 120 seconds, the Lyse-It^®^ slides gold coating had cracked. While no gold itself went into solution, several trials of used slides showed identical heating as compared to standard microwave heating. This suggests that the slides are damaged after a single use and cannot be reused (**S3 Fig**).

## Discussion

In rapid diagnostic settings, it is imperative that there is also a rapid accompanying sample preparation protocol that is not cost and time prohibitive. Most pathogenic sample preparation techniques today involve a two-step process of bacterial cellular lysis followed be DNA/RNA fragmentation and recovery; many even require the use of expensive equipment, such as centrifuges and bead beaters. In this paper, we have demonstrated a one step process for Gram-negative and -positive bacterial cellular lysis, DNA fragmentation, and protein extraction called Lyse-it^®^. Lyse-It^®^ was applied to *L*. *monocytogenes* and *V*. *cholerae*, validating its use on various forms of pathogenic organisms.

We have shown that the Lyse-It^®^ technology increases the overall temperature of the sample contributing to more efficient cellular lysis, although a thermal mechanism is not believed to be the dominant mechanism for biomolecule degradation. TEM images and DLS studies show that as microwave power is increased, cellular disruption increases, more so than standard microwave heating. Significant disruption of the cellular membrane is seen in *V*. *cholerae* with an applied microwave irradiation of 30% power for 60 seconds. Conversely, an increased power (50%) is required for the Gram-positive organism *L*. *monocytogenes*, which is not surprising due to the different architecture of the cell membrane [26, 27].

Consistent with other reports on cellular lysis and DNA fragmentation for various bacteria [12, 21, 25, 28–32], we observe an increase in DNA fragmentation as microwave irradiation and irradiation time increases with Lyse-It^®^. As demonstrated by the *L*. *monocytogenes* bioanalyzer mini gel results, as the irradiation power and/or time increases, genomic DNA fragmentation progressively increases (cuts to shorter and then shorter pieces) eventually resulting in low total concentrations (pg/μL) of short DNA fragments. DNA fragments around and below 500 bp allow for pathogen genomic DNA identification on platforms like qPCR, and as shown in **Fig 4**, Lyse-It^®^ conditions of 30% power. 30 seconds, already results in a significant amount of released DNA.

Following confirmation of bacterial cellular lysis and DNA fragmentation, the genomic DNA from *L*. *monocytogenes* and *V*. *cholerae* was purified, amplified, and detected using qPCR. Consistent with an increase in cellular lysis and intracellular component release as microwave irradiation increases, the amount of DNA detected also increased. These results further validate that genomic DNA released and fragmented from both Gram-negative and -positive organisms can be detected and quantified on a qPCR platform.

Further investigation of released cellular components led to the experimentation of microwave effects on both released (early lysing times) and degraded proteins (later lysing times). Conventional heating methods, which are widely used today for cellular lysis, show a consistent release of proteins with minimal degradation. Conversely, the Lyse-It^®^ technology shows a much greater increase in protein release and subsequent protein degradation at later times. This “tunability” of being able to either simply release and collect protein for applications such as proteomics, or indeed degrade protein at later lysing times, is simply a factor of energy introduced into the sample, where energy = time (secs) multiplied by Microwave power (Watts). Interestingly, we see the same influence on DNA also, where at early lysing times, DNA/RNA is intact and at later times, fragmented. This potentially allows the Lyse-It^®^ technology to be used for whole genome sequencing, where intact DNA is needed and can be collected at early lysing times versus fragmented DNA at later lysing times, which as we have shown is ideal for qPCR. Future studies from our laboratory will also look at detecting specific released proteins using Lyse-It^®^, at early times in the lysing process when biomolecules are still intact. The sequence of events during Lyse-It^®^ based lysing, as determined from this study is shown in **S4 Fig**.

Lyse-It^®^ can serve as a tunable platform for cellular lysis and DNA fragmentation potentially for a wide variety of DNA detection platforms. While beyond the scope of this manuscript, we aim to show Lyse-It^®^ effects on RNA and nucleases as well as elucidate the non-thermal mechanism for DNA/RNA fragmentation in the future.

## Supporting information

S1 Fig **A:** Lyse-It^®^ slide composed of two vapor deposited gold equatorial 12.3 mm triangles in a “bow-tie” configuration. **B:** Lyse-It^®^ slide held on a microwave sample-mounting block.
(TIF)

S2 Fig Ethidium bromide stained gels of room temperature osmotically lysed *V*. *cholerae* (**A**) and *L*. *monocytogenes* (**B**). L5: 500 bp ladder, L1: 100 bp ladder (**A**) Lane 1: 10 min, Lanes 2–4: 1–3 hours, Lane 5: 24 hours, Lane 6: 48 hours, Lane 7: 3.5 days, Lanes 8–10: 1, 2, 2.5 weeks. (**B**) Lane 1: 10 minutes, Lanes 2–4: 1, 2, 2.5 hours, Lanes 5–7: 1, 2, 2.5 weeks. DNA fragmentation can be seen on a standard gel after about 48 hours for *V*. *cholerae* and 1 week for *L*. *monocytogenes*.
(TIF)

S3 Fig After a single use, the Lyse-It^®^ slides cannot be reused. The heating profile for *used* Lyse-It^®^ slides is similar to simple microwave heating without the use of the Lyse-It^®^ slides.
(TIF)

S4 Fig Sequence of events during microwave-based lysing using Lyse-It^®^. (DOCX)
