# Supplementary figures and images for: Rapid sample preparation with Lyse-It® for Listeria monocytogenes and Vibrio cholerae
Source: PLoS One. 2018 Jul 25;13(7):e0201070. doi: 10.1371/journal.pone.0201070 (PMC6059484; doi:10.1371/journal.pone.0201070)

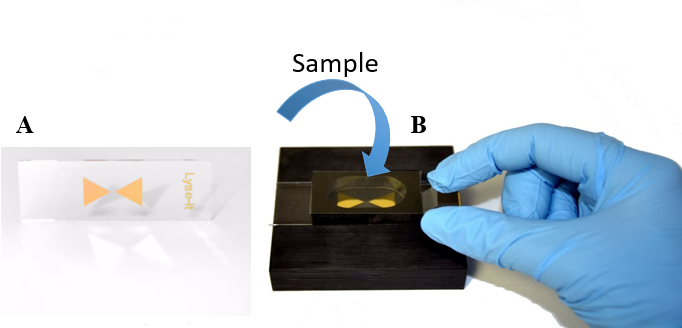

Supplement: S1 Fig — A: Lyse-It® slide composed of two vapor deposited gold equatorial 12.3 mm triangles in a “bow-tie” configuration. B: Lyse-It® slide held on a microwave sample-mounting block. (TIF) [file pone.0201070.s001.tif]

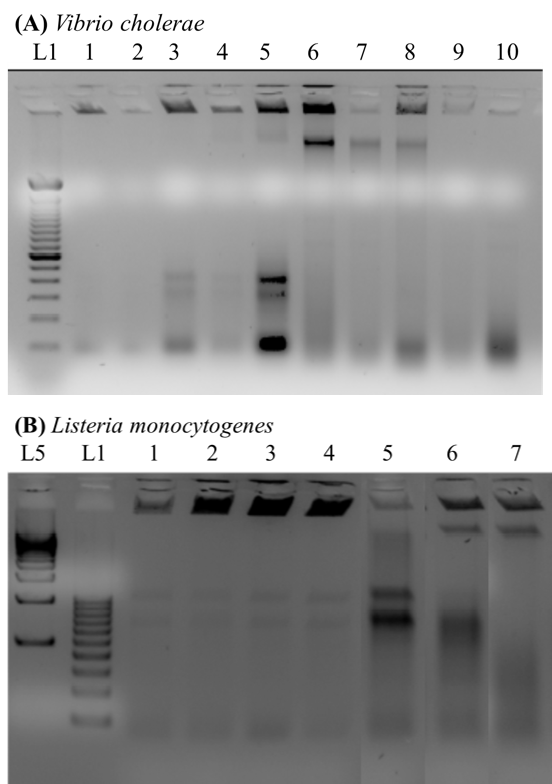

Supplement: S2 Fig — Ethidium bromide stained gels of room temperature osmotically lysed V. cholerae (A) and L. monocytogenes (B). L5: 500 bp ladder, L1: 100 bp ladder (A) Lane 1: 10 min, Lanes 2–4: 1–3 hours, Lane 5: 24 hours, Lane 6: 48 hours, Lane 7: 3.5 days, Lanes 8–10: 1, 2, 2.5 weeks. (B) Lane 1: 10 minutes, Lanes 2–4: 1, 2, 2.5 hours, Lanes 5–7: 1, 2, 2.5 weeks. DNA fragmentation can be seen on a standard gel after about 48 hours for V. cholerae and 1 week for L. monocytogenes. (TIF) [file pone.0201070.s002.tif]

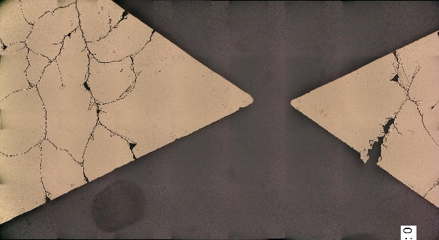

Supplement: S3 Fig — The heating profile for used Lyse-It® slides is similar to simple microwave heating without the use of the Lyse-It® slides. (TIF) [file pone.0201070.s003.tif]

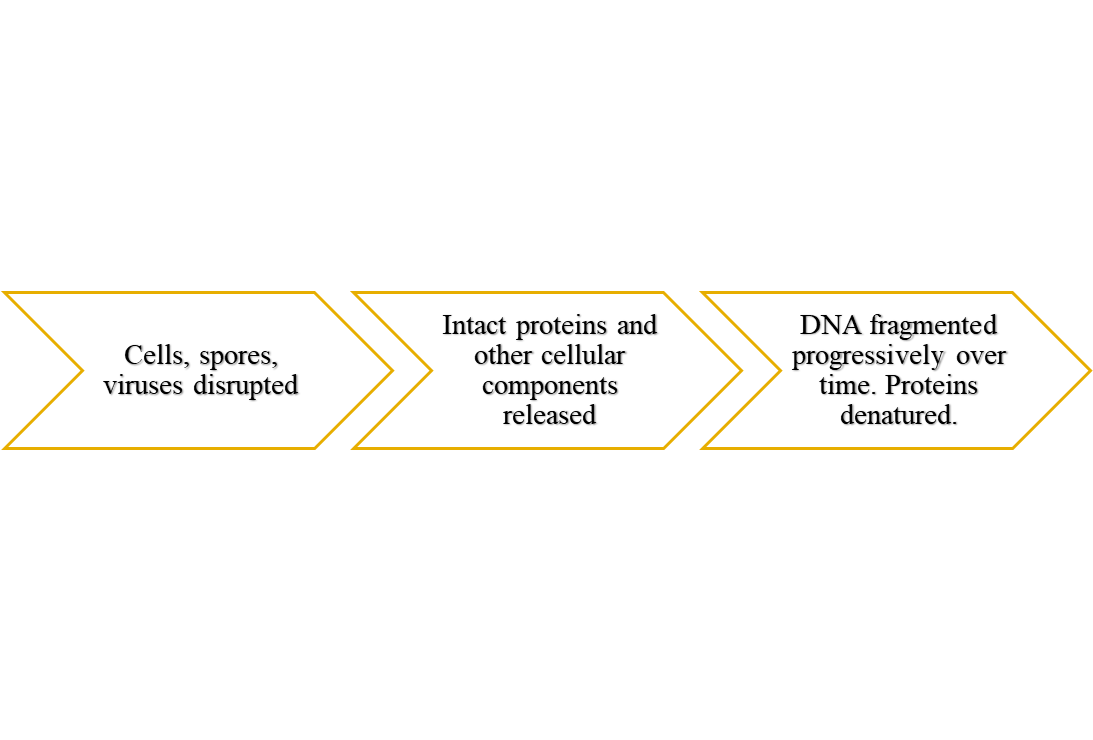

Supplement: S4 Fig — (DOCX) [file pone.0201070.s004.docx]
